# Ethyl 2-cyano-5-oxo-5-(thio­phen-2-yl)-3-(3,4,5-trimeth­oxy­phen­yl)penta­noate

**DOI:** 10.1107/S1600536812044522

**Published:** 2012-11-03

**Authors:** M. Prabhuswamy, S. Madan Kumar, K. R. Raghavendra, M. M. M. Abdoh, S. Shashikanth, N. K. Lokanath

**Affiliations:** a Department of Studies in Physics, Manasagangotri, University of Mysore, Mysore 570 006, India; b Department of Studies in Chemistry, University of Mysore, Manasagangotri, Mysore 570 006, India; c Department of Physics, Faculty of Science, An Najah National University, Nabtus West Bank, Palestinian Territories

## Abstract

In the title compound, C_21_H_23_NO_6_S, the dihedral angle between the thio­pene and benzene rings is 88.66 (6)°. In the crystal, mol­ecules are connected by C—H⋯N and C—H⋯O hydrogen bonds, forming a tape along [10-1]. In addition, C—H⋯π and π–π stacking [centroid–centroid distance = 3.879 (2) Å between the thio­phene rings] inter­actions are observed.

## Related literature

For applications of thio­phenes, see: Günther & Steinmetz (1963 ▶). For a similar structure, see: Harrison *et al.* (2010 ▶).
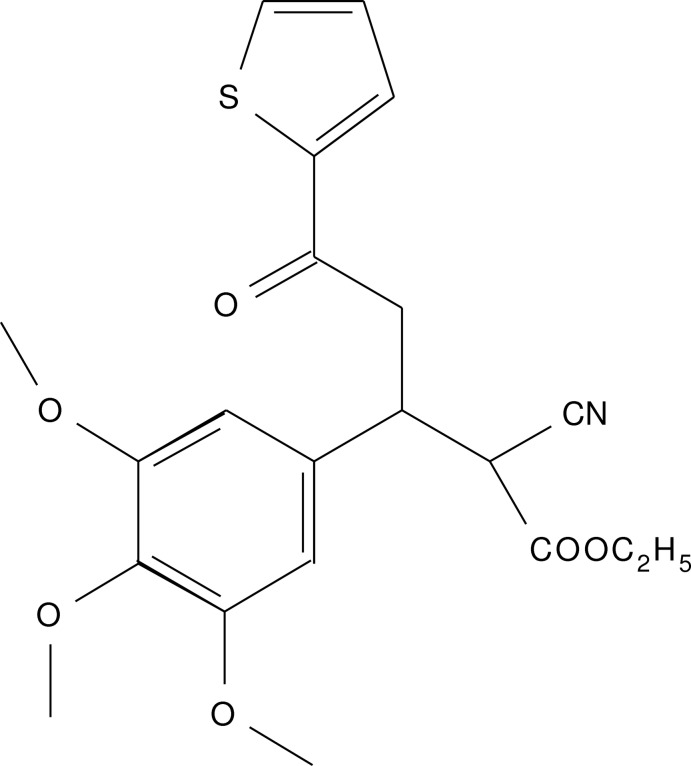


## Experimental

### 

#### Crystal data

C_21_H_23_NO_6_S
*M* *_r_* = 417.47
Triclinic, 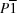

*a* = 8.4308 (5) Å
*b* = 10.5025 (6) Å
*c* = 12.3059 (6) Å
α = 98.530 (2)°
β = 107.950 (2)°
γ = 97.966 (3)°
*V* = 1005.26 (10) Å^3^
*Z* = 2
Mo *K*α radiation
μ = 0.20 mm^−1^
*T* = 296 K
0.22 × 0.20 × 0.19 mm

#### Data collection

Oxford Diffraction Xcalibur Eos diffractometer
16712 measured reflections
3965 independent reflections
3498 reflections with *I* > 2σ(*I*)
*R*_int_ = 0.027

#### Refinement

*R*[*F* ^2^ > 2σ(*F* ^2^)] = 0.032
*wR*(*F* ^2^) = 0.080
*S* = 1.04
3965 reflections
266 parameters
H-atom parameters constrained
Δρ_max_ = 0.30 e Å^−3^
Δρ_min_ = −0.29 e Å^−3^

### 

Data collection: *CrysAlis PRO* (Oxford Diffraction, 2009 ▶); cell refinement: *CrysAlis PRO*; data reduction: *CrysAlis PRO*; program(s) used to solve structure: *SHELXS97* (Sheldrick, 2008 ▶); program(s) used to refine structure: *SHELXL97* (Sheldrick, 2008 ▶); molecular graphics: *Mercury* (Macrae *et al.*, 2006 ▶); software used to prepare material for publication: *Mercury*.

## Supplementary Material

Crystal structure: contains datablock(s) global, I. DOI: 10.1107/S1600536812044522/is5211sup1.cif

Structure factors: contains datablock(s) I. DOI: 10.1107/S1600536812044522/is5211Isup2.hkl

Supplementary material file. DOI: 10.1107/S1600536812044522/is5211Isup3.cml

Additional supplementary materials:  crystallographic information; 3D view; checkCIF report

## Figures and Tables

**Table 1:** Hydrogen-bond geometry (Å, °) *Cg*2 is the centroid of the C8–C13 ring.

*D*—H⋯*A*	*D*—H	H⋯*A*	*D*⋯*A*	*D*—H⋯*A*
C3—H3⋯N1^i^	0.93	2.49	3.368 (2)	157
C18—H18*B*⋯O2^ii^	0.96	2.56	3.385 (2)	143
C17—H17*A*⋯*Cg*2^iii^	0.96	2.85	3.6327 (16)	139

Symmetry codes: (i) 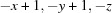; (ii) 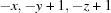; (iii) 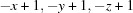.

**Fig. 1.**
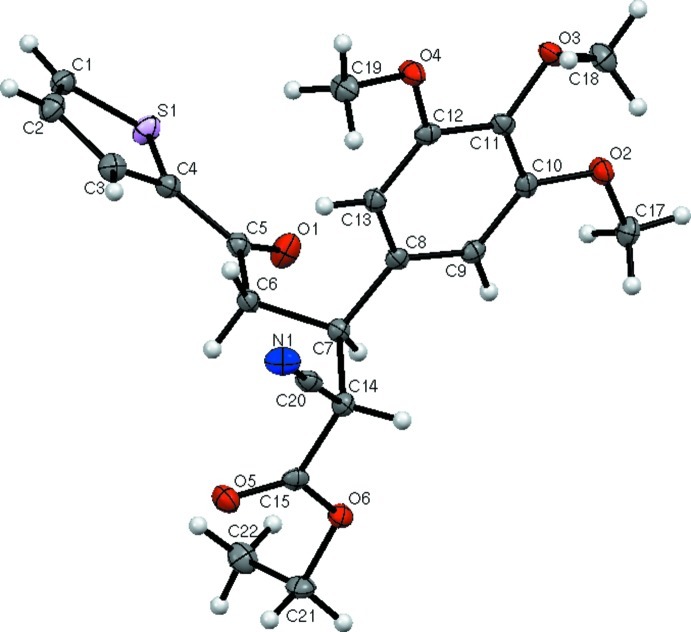
An ORTEP diagram of the title compound with 50% probability ellipsoids.

**Fig. 2.**
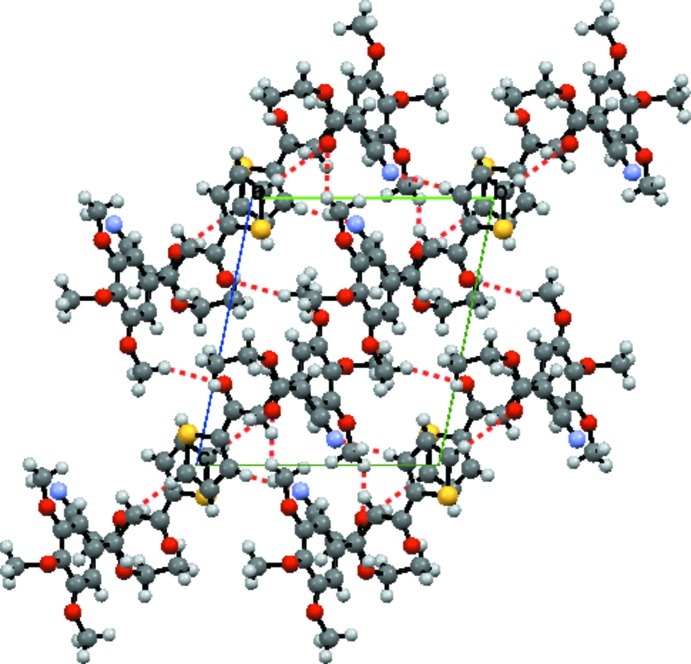
A packing diagram of the title compound, viewed along the a-axis. C—H···N and C—H···O hydrogen bonds are indicated by dashed lines.

## supplementary crystallographic information

### Comment

Thiophenes have importance to give cycloaddition products with carbenes (Günther *et al.*,1963). In the title molecule, C_21_H_23_NO_6_S (Fig. 1.), the thiopene ring is basically planar and its geometry is similar to (2*E*)-3-(1,3-benzodioxol-5-yl)-1-(3-bromo-2-thienyl)prop-2-en-1-one (Harrison *et al.*, 2010). The dihedral angle between the thiopene ring and the trimethoxyphenyl unit is 88.66 (6)°, which signifies the thiopene ring is almost perpendicular to the trimethoxyphenyl unit. The molecules are connected by C—H···N and C—H···O interactions (Table 1) into a tape structure (Fig. 2). In addition, the crystal is stabilized with π–π stacking interactions between thiophene rings, related by unit translation along the *a* axis with the distance of 3.879 (2) Å [*-x*+1, *-y*+1, *-z*+1]. Also, short contacts C—H···π (Table 1) and C—O···π with a distance of 3.733 (1) Å [87.10 (9)°] [*x*+1, *y*, *z*] are present.

### Experimental

Freshly distilled ethyl cyanoacetate (8.5 g, 0.72 mol) was added in to a stirred suspension of powdered sodium (2.51 g, 0.109 mol) in dry benzene (40 ml) at room temperature. To this mixture Chalcone-1{3-(benzo[1,3] dioxol-5-yl)-1(thiophene-2-yl)prop-2-ene-1-one} was added and stirred for 36 hrs, at room temperature. Salts were filtered off, the filterate was with 5% NaOH (100 ml), brine solution (100 ml), and dried over anhydrous sodium sulfate. Concentration of the solvent furnished crude product, which was purified by column chromatography using benzene-ethyl acetate (8:2) as eluent gave ethyl 2-cyano-5-oxo-5-(thiophen-2-yl)-3-(3,4,5-trimethoxyphenyl)pentanoate as pale yellow oily product in 78% yield (14.5 g). Recrystallization with benzene led to the formation of the crystals.

### Refinement

All H atoms were positioned geometrically (C—H = 0.93–0.98 Å) and allowed to ride on their parent atoms, with *U*_iso_(H) = 1.2*U*_eq_(aromatic C) or 1.5*U*_eq_(methyl C). One bad reflection (-2 2 0) has been omitted in the final refinement.

### Crystal data

**Table:**

C_21_H_23_NO_6_S	*Z* = 2
*M**_r_* = 417.47	*F*(000) = 440
Triclinic, *P*1	*D*_x_ = 1.379 Mg m^−^^3^
Hall symbol: -P 1	Mo *K*α radiation, λ = 0.71073 Å
*a* = 8.4308 (5) Å	Cell parameters from 3965 reflections
*b* = 10.5025 (6) Å	θ = 1.8–26.0°
*c* = 12.3059 (6) Å	µ = 0.20 mm^−^^1^
α = 98.530 (2)°	*T* = 296 K
β = 107.950 (2)°	Block, white
γ = 97.966 (3)°	0.22 × 0.20 × 0.19 mm
*V* = 1005.26 (10) Å^3^	

### Data collection

**Table:**

Oxford Diffraction Xcalibur Eos diffractometer	3498 reflections with *I* > 2σ(*I*)
Radiation source: fine-focus sealed tube	*R*_int_ = 0.027
Graphite monochromator	θ_max_ = 26.0°, θ_min_ = 1.8°
Detector resolution: 16.0839 pixels mm^-1^	*h* = −10→10
ω scans	*k* = −12→12
16712 measured reflections	*l* = −15→15
3965 independent reflections	

### Refinement

**Table:**

Refinement on *F*^2^	Primary atom site location: structure-invariant direct methods
Least-squares matrix: full	Secondary atom site location: difference Fourier map
*R*[*F*^2^ > 2σ(*F*^2^)] = 0.032	Hydrogen site location: inferred from neighbouring sites
*wR*(*F*^2^) = 0.080	H-atom parameters constrained
*S* = 1.04	*w* = 1/[σ^2^(*F*_o_^2^) + (0.0336*P*)^2^ + 0.4919*P*] where *P* = (*F*_o_^2^ + 2*F*_c_^2^)/3
3965 reflections	(Δ/σ)_max_ < 0.001
266 parameters	Δρ_max_ = 0.30 e Å^−^^3^
0 restraints	Δρ_min_ = −0.29 e Å^−^^3^

### Special details

**Table:**

Experimental. IR: 2249 cm^-1^ (CN), 1758 cm^-1^ (esterCO). ^1^H NMR: 400 MHz (CDCl_3_) *δ*: 7.8 (1*H*, s), 7.3 (1*H*, s), 7.15 (1*H*, s), 6.6 (2*H*, s), 4.3 (1*H*, m), 4.2 (2*H*, q), 3.91 (1*H*, d), 3.88(6*H*, s), 3.8 (3*H*, s), 3.58 (2*H*, d), 1.23 (3*H*, t). MS: (*M*^+^+1): 417.114.
Geometry. Bond distances, angles *etc*. have been calculated using the rounded fractional coordinates. All su's are estimated from the variances of the (full) variance-covariance matrix. The cell e.s.d.'s are taken into account in the estimation of distances, angles and torsion angles
Refinement. Refinement on *F*^2^ for ALL reflections except those flagged by the user for potential systematic errors. Weighted *R*-factors *wR* and all goodnesses of fit *S* are based on *F*^2^, conventional *R*-factors *R* are based on *F*, with *F* set to zero for negative *F*^2^. The observed criterion of *F*^2^ > σ(*F*^2^) is used only for calculating -*R*-factor-obs *etc*. and is not relevant to the choice of reflections for refinement. *R*-factors based on *F*^2^ are statistically about twice as large as those based on *F*, and *R*-factors based on ALL data will be even larger.

### Fractional atomic coordinates and isotropic or equivalent isotropic displacement parameters (Å^2^)

**Table:**

	*x*	*y*	*z*	*U*_iso_*/*U*_eq_	
S1	0.28565 (5)	1.06346 (3)	0.11584 (3)	0.0210 (1)	
O1	0.51411 (14)	0.95781 (10)	0.30335 (8)	0.0242 (3)	
O2	0.30973 (12)	0.61342 (9)	0.55116 (8)	0.0190 (3)	
O3	0.05123 (12)	0.47024 (9)	0.37057 (8)	0.0185 (3)	
O4	0.07586 (12)	0.41627 (10)	0.15717 (8)	0.0196 (3)	
O5	0.97151 (12)	0.73365 (10)	0.20324 (8)	0.0209 (3)	
O6	1.01797 (12)	0.78955 (9)	0.39592 (8)	0.0170 (3)	
N1	0.65206 (15)	0.45085 (12)	0.09731 (11)	0.0234 (4)	
C1	0.18369 (17)	1.03613 (14)	−0.03091 (12)	0.0200 (4)	
C2	0.21615 (18)	0.92745 (14)	−0.08935 (12)	0.0207 (4)	
C3	0.32723 (18)	0.86473 (14)	−0.01290 (12)	0.0193 (4)	
C4	0.37613 (17)	0.92704 (13)	0.10141 (12)	0.0160 (4)	
C5	0.49077 (17)	0.89505 (13)	0.20599 (11)	0.0161 (4)	
C6	0.58474 (17)	0.78544 (13)	0.18750 (11)	0.0156 (4)	
C7	0.63629 (16)	0.72138 (12)	0.29378 (11)	0.0139 (3)	
C8	0.48318 (16)	0.64602 (12)	0.31321 (11)	0.0140 (3)	
C9	0.47425 (17)	0.66368 (13)	0.42533 (11)	0.0146 (3)	
C10	0.33230 (17)	0.60120 (13)	0.44517 (11)	0.0152 (4)	
C11	0.19780 (17)	0.52151 (13)	0.35220 (11)	0.0150 (4)	
C12	0.21070 (16)	0.49990 (13)	0.24072 (11)	0.0153 (3)	
C13	0.35232 (17)	0.56266 (13)	0.22062 (11)	0.0151 (4)	
C14	0.77024 (16)	0.63512 (13)	0.28577 (11)	0.0147 (4)	
C15	0.93232 (17)	0.72248 (13)	0.28766 (11)	0.0149 (3)	
C17	0.44421 (18)	0.69491 (14)	0.64868 (11)	0.0201 (4)	
C18	0.03823 (19)	0.33577 (14)	0.37964 (13)	0.0224 (4)	
C19	0.09595 (18)	0.37209 (14)	0.04739 (12)	0.0216 (4)	
C20	0.70638 (17)	0.53120 (13)	0.17950 (12)	0.0168 (4)	
C21	1.17352 (17)	0.88197 (14)	0.41028 (12)	0.0202 (4)	
C22	1.1345 (2)	1.00956 (14)	0.37958 (13)	0.0243 (4)	
H1	0.11360	1.08950	−0.06710	0.0240*	
H2	0.17080	0.89800	−0.16980	0.0250*	
H3	0.36310	0.78930	−0.03780	0.0230*	
H6A	0.68590	0.82030	0.17130	0.0190*	
H6B	0.51270	0.71930	0.12010	0.0190*	
H7	0.69170	0.79260	0.36220	0.0170*	
H9	0.56340	0.71740	0.48710	0.0180*	
H13	0.35970	0.54910	0.14610	0.0180*	
H14	0.79750	0.59380	0.35390	0.0180*	
H17A	0.54680	0.66130	0.65800	0.0300*	
H17B	0.41450	0.69530	0.71800	0.0300*	
H17C	0.46160	0.78280	0.63550	0.0300*	
H18A	0.03270	0.28360	0.30710	0.0340*	
H18B	−0.06280	0.30720	0.39710	0.0340*	
H18C	0.13600	0.32590	0.44090	0.0340*	
H19A	0.11030	0.44550	0.01080	0.0320*	
H19B	−0.00320	0.30890	−0.00200	0.0320*	
H19C	0.19420	0.33230	0.05960	0.0320*	
H21A	1.23170	0.84380	0.36060	0.0240*	
H21B	1.24880	0.89780	0.49050	0.0240*	
H22A	1.06580	0.99480	0.29880	0.0360*	
H22B	1.23870	1.06950	0.39310	0.0360*	
H22C	1.07400	1.04620	0.42710	0.0360*	

### Atomic displacement parameters (Å^2^)

**Table:**

	*U*^11^	*U*^22^	*U*^33^	*U*^12^	*U*^13^	*U*^23^
S1	0.0241 (2)	0.0211 (2)	0.0196 (2)	0.0112 (2)	0.0066 (1)	0.0051 (1)
O1	0.0334 (6)	0.0209 (5)	0.0182 (5)	0.0120 (4)	0.0065 (4)	0.0025 (4)
O2	0.0206 (5)	0.0222 (5)	0.0150 (5)	0.0019 (4)	0.0084 (4)	0.0029 (4)
O3	0.0158 (5)	0.0190 (5)	0.0255 (5)	0.0046 (4)	0.0120 (4)	0.0068 (4)
O4	0.0160 (5)	0.0241 (5)	0.0162 (5)	−0.0015 (4)	0.0058 (4)	0.0008 (4)
O5	0.0206 (5)	0.0233 (5)	0.0196 (5)	0.0014 (4)	0.0101 (4)	0.0021 (4)
O6	0.0154 (5)	0.0173 (5)	0.0168 (5)	−0.0002 (4)	0.0046 (4)	0.0036 (4)
N1	0.0211 (6)	0.0187 (6)	0.0283 (7)	0.0050 (5)	0.0073 (5)	−0.0007 (5)
C1	0.0162 (7)	0.0214 (7)	0.0230 (7)	0.0041 (6)	0.0047 (6)	0.0096 (6)
C2	0.0208 (7)	0.0197 (7)	0.0185 (7)	0.0020 (6)	0.0031 (6)	0.0043 (5)
C3	0.0203 (7)	0.0151 (7)	0.0215 (7)	0.0027 (5)	0.0065 (6)	0.0029 (5)
C4	0.0148 (6)	0.0136 (7)	0.0211 (7)	0.0038 (5)	0.0071 (5)	0.0049 (5)
C5	0.0164 (7)	0.0140 (6)	0.0189 (7)	0.0017 (5)	0.0070 (5)	0.0049 (5)
C6	0.0158 (7)	0.0156 (7)	0.0184 (6)	0.0039 (5)	0.0083 (5)	0.0061 (5)
C7	0.0146 (6)	0.0122 (6)	0.0155 (6)	0.0029 (5)	0.0056 (5)	0.0030 (5)
C8	0.0143 (6)	0.0123 (6)	0.0184 (6)	0.0061 (5)	0.0069 (5)	0.0057 (5)
C9	0.0150 (6)	0.0125 (6)	0.0159 (6)	0.0039 (5)	0.0038 (5)	0.0035 (5)
C10	0.0187 (7)	0.0151 (7)	0.0158 (6)	0.0075 (5)	0.0084 (5)	0.0056 (5)
C11	0.0143 (6)	0.0138 (6)	0.0207 (7)	0.0059 (5)	0.0084 (5)	0.0064 (5)
C12	0.0141 (6)	0.0136 (6)	0.0183 (6)	0.0050 (5)	0.0045 (5)	0.0036 (5)
C13	0.0166 (7)	0.0162 (7)	0.0153 (6)	0.0060 (5)	0.0073 (5)	0.0047 (5)
C14	0.0153 (7)	0.0134 (6)	0.0159 (6)	0.0034 (5)	0.0056 (5)	0.0034 (5)
C15	0.0138 (6)	0.0134 (6)	0.0186 (6)	0.0064 (5)	0.0054 (5)	0.0036 (5)
C17	0.0239 (7)	0.0224 (7)	0.0139 (6)	0.0047 (6)	0.0063 (5)	0.0034 (5)
C18	0.0232 (7)	0.0192 (7)	0.0263 (7)	0.0006 (6)	0.0112 (6)	0.0066 (6)
C19	0.0207 (7)	0.0232 (7)	0.0176 (7)	0.0005 (6)	0.0063 (6)	−0.0016 (6)
C20	0.0132 (6)	0.0159 (7)	0.0241 (7)	0.0055 (5)	0.0083 (5)	0.0061 (6)
C21	0.0133 (7)	0.0214 (7)	0.0220 (7)	−0.0010 (6)	0.0027 (5)	0.0033 (6)
C22	0.0264 (8)	0.0184 (7)	0.0273 (8)	0.0003 (6)	0.0105 (6)	0.0033 (6)

### Geometric parameters (Å, º)

**Table:**

S1—C1	1.7040 (14)	C14—C15	1.528 (2)
S1—C4	1.7254 (15)	C14—C20	1.4734 (19)
O1—C5	1.2219 (16)	C21—C22	1.497 (2)
O2—C10	1.3653 (16)	C1—H1	0.9300
O2—C17	1.4303 (17)	C2—H2	0.9300
O3—C11	1.3755 (18)	C3—H3	0.9300
O3—C18	1.4249 (18)	C6—H6A	0.9700
O4—C12	1.3623 (17)	C6—H6B	0.9700
O4—C19	1.4304 (17)	C7—H7	0.9800
O5—C15	1.1996 (17)	C9—H9	0.9300
O6—C15	1.3342 (16)	C13—H13	0.9300
O6—C21	1.4651 (18)	C14—H14	0.9800
N1—C20	1.1397 (19)	C17—H17A	0.9600
C1—C2	1.364 (2)	C17—H17B	0.9600
C2—C3	1.417 (2)	C17—H17C	0.9600
C3—C4	1.368 (2)	C18—H18A	0.9600
C4—C5	1.4679 (19)	C18—H18B	0.9600
C5—C6	1.514 (2)	C18—H18C	0.9600
C6—C7	1.5309 (18)	C19—H19A	0.9600
C7—C8	1.521 (2)	C19—H19B	0.9600
C7—C14	1.559 (2)	C19—H19C	0.9600
C8—C9	1.3913 (18)	C21—H21A	0.9700
C8—C13	1.3941 (19)	C21—H21B	0.9700
C9—C10	1.390 (2)	C22—H22A	0.9600
C10—C11	1.3959 (19)	C22—H22B	0.9600
C11—C12	1.3971 (18)	C22—H22C	0.9600
C12—C13	1.391 (2)		
			
C1—S1—C4	91.52 (7)	C4—C3—H3	124.00
C10—O2—C17	117.05 (11)	C5—C6—H6A	109.00
C11—O3—C18	113.37 (11)	C5—C6—H6B	109.00
C12—O4—C19	117.02 (11)	C7—C6—H6A	109.00
C15—O6—C21	115.87 (11)	C7—C6—H6B	109.00
S1—C1—C2	112.56 (11)	H6A—C6—H6B	108.00
C1—C2—C3	111.94 (13)	C6—C7—H7	107.00
C2—C3—C4	112.82 (13)	C8—C7—H7	107.00
S1—C4—C3	111.16 (11)	C14—C7—H7	107.00
S1—C4—C5	119.12 (10)	C8—C9—H9	120.00
C3—C4—C5	129.71 (13)	C10—C9—H9	120.00
O1—C5—C4	121.25 (13)	C8—C13—H13	120.00
O1—C5—C6	121.58 (12)	C12—C13—H13	120.00
C4—C5—C6	117.12 (11)	C7—C14—H14	109.00
C5—C6—C7	112.22 (11)	C15—C14—H14	109.00
C6—C7—C8	112.13 (11)	C20—C14—H14	109.00
C6—C7—C14	110.99 (11)	O2—C17—H17A	109.00
C8—C7—C14	112.42 (11)	O2—C17—H17B	109.00
C7—C8—C9	118.67 (12)	O2—C17—H17C	109.00
C7—C8—C13	121.01 (12)	H17A—C17—H17B	109.00
C9—C8—C13	120.31 (13)	H17A—C17—H17C	109.00
C8—C9—C10	120.15 (12)	H17B—C17—H17C	109.00
O2—C10—C9	124.82 (12)	O3—C18—H18A	109.00
O2—C10—C11	115.26 (13)	O3—C18—H18B	109.00
C9—C10—C11	119.90 (12)	O3—C18—H18C	109.00
O3—C11—C10	119.50 (12)	H18A—C18—H18B	110.00
O3—C11—C12	120.84 (12)	H18A—C18—H18C	109.00
C10—C11—C12	119.60 (13)	H18B—C18—H18C	109.00
O4—C12—C11	115.00 (12)	O4—C19—H19A	109.00
O4—C12—C13	124.50 (12)	O4—C19—H19B	109.00
C11—C12—C13	120.51 (12)	O4—C19—H19C	109.00
C8—C13—C12	119.43 (12)	H19A—C19—H19B	109.00
C7—C14—C15	109.30 (11)	H19A—C19—H19C	109.00
C7—C14—C20	111.98 (11)	H19B—C19—H19C	110.00
C15—C14—C20	109.62 (11)	O6—C21—H21A	109.00
O5—C15—O6	125.34 (13)	O6—C21—H21B	109.00
O5—C15—C14	124.81 (12)	C22—C21—H21A	109.00
O6—C15—C14	109.74 (11)	C22—C21—H21B	109.00
N1—C20—C14	177.91 (16)	H21A—C21—H21B	108.00
O6—C21—C22	111.19 (12)	C21—C22—H22A	109.00
S1—C1—H1	124.00	C21—C22—H22B	109.00
C2—C1—H1	124.00	C21—C22—H22C	110.00
C1—C2—H2	124.00	H22A—C22—H22B	109.00
C3—C2—H2	124.00	H22A—C22—H22C	109.00
C2—C3—H3	124.00	H22B—C22—H22C	109.00
			
C4—S1—C1—C2	0.13 (13)	C14—C7—C8—C9	100.64 (14)
C1—S1—C4—C3	−0.09 (13)	C14—C7—C8—C13	−80.57 (15)
C1—S1—C4—C5	179.01 (12)	C6—C7—C14—C15	63.27 (13)
C17—O2—C10—C9	−1.2 (2)	C6—C7—C14—C20	−58.39 (15)
C17—O2—C10—C11	−179.64 (12)	C8—C7—C14—C15	−170.23 (10)
C18—O3—C11—C10	−101.99 (15)	C8—C7—C14—C20	68.11 (14)
C18—O3—C11—C12	81.04 (16)	C7—C8—C9—C10	176.92 (12)
C19—O4—C12—C11	−168.81 (12)	C13—C8—C9—C10	−1.9 (2)
C19—O4—C12—C13	12.0 (2)	C7—C8—C13—C12	−177.12 (12)
C21—O6—C15—O5	−1.1 (2)	C9—C8—C13—C12	1.7 (2)
C21—O6—C15—C14	−177.57 (11)	C8—C9—C10—O2	−178.97 (13)
C15—O6—C21—C22	82.45 (15)	C8—C9—C10—C11	−0.6 (2)
S1—C1—C2—C3	−0.14 (17)	O2—C10—C11—O3	4.74 (19)
C1—C2—C3—C4	0.1 (2)	O2—C10—C11—C12	−178.24 (12)
C2—C3—C4—S1	0.03 (18)	C9—C10—C11—O3	−173.80 (13)
C2—C3—C4—C5	−178.95 (15)	C9—C10—C11—C12	3.2 (2)
S1—C4—C5—O1	5.0 (2)	O3—C11—C12—O4	−5.74 (19)
S1—C4—C5—C6	−172.23 (10)	O3—C11—C12—C13	173.52 (13)
C3—C4—C5—O1	−176.11 (16)	C10—C11—C12—O4	177.29 (12)
C3—C4—C5—C6	6.7 (2)	C10—C11—C12—C13	−3.5 (2)
O1—C5—C6—C7	27.39 (19)	O4—C12—C13—C8	−179.79 (13)
C4—C5—C6—C7	−155.42 (12)	C11—C12—C13—C8	1.0 (2)
C5—C6—C7—C8	67.74 (14)	C7—C14—C15—O5	−100.86 (16)
C5—C6—C7—C14	−165.60 (11)	C7—C14—C15—O6	75.65 (13)
C6—C7—C8—C9	−133.47 (13)	C20—C14—C15—O5	22.22 (19)
C6—C7—C8—C13	45.31 (17)	C20—C14—C15—O6	−161.28 (11)

### Hydrogen-bond geometry (Å, º)

*Cg*2 is the centroid of the C8–C13 ring.

**Table:**

*D*—H···*A*	*D*—H	H···*A*	*D*···*A*	*D*—H···*A*
C3—H3···N1^i^	0.93	2.49	3.368 (2)	157
C18—H18*B*···O2^ii^	0.96	2.56	3.385 (2)	143
C17—H17*A*···*Cg*2^iii^	0.96	2.85	3.6327 (16)	139

Symmetry codes: (i) −*x*+1, −*y*+1, −*z*; (ii) −*x*, −*y*+1, −*z*+1; (iii) −*x*+1, −*y*+1, −*z*+1.
